# Health and educational success in adolescents: a longitudinal study

**DOI:** 10.1186/s12889-015-1966-0

**Published:** 2015-07-07

**Authors:** Idunn Brekke

**Affiliations:** Department of Nursing – Faculty of Health Sciences, Oslo and Akershus University College of Applied Sciences, Postbox 4 St. Olavs plass, N-0130 Oslo, Norway

**Keywords:** Infant health, Adolescent health, Health selection, Socioeconomic status, Higher education

## Abstract

**Background:**

Health in childhood and adolescence is a matter of contention. This article examines how infant and adolescent health act together with parental SES, health-related behaviour and academic factors to generate differences in the early life course with regard to later enrolment in higher education.

**Methods:**

We used a questionnaire on health, The Oslo Health Study, which was linked to register data that provided detailed information on educational outcomes over time; and the Medical Birth Registry of Norway, which provided information on health at birth.

**Results:**

It was found in the unadjusted results that infant health measures had a positive association with enrolment in higher education. After adjustment for adolescent health, there was still evidence that infant health are associated with enrolment in higher education. However, this association disappeared when parental socio-economic status (SES) was included in the model. Health in adolescents remains a significant and strong predictor of enrolment in higher education after adjusting for parental SES. However, the relationship between adolescent health and enrolment in higher education was reduced and became nonsignificant when adjustments were made to the health behaviour of the adolescents and their relationship with their families. Future educational expectations and good grades in grade 10 are strong predictors of enrolment in higher education.

**Conclusions:**

There are lower odds of enrolment in higher education for infants of low birthweight. However, this result seems to reflect the fact that parental SES correlate with both infant health and enrolment in higher education. Adolescent health are associated with enrolment in higher education, even after adjusting for parental SES. However, a considerable proportion of this association seems to be attributable to health-related behaviour and the relationship of the adolescent with his or her family.

## Background

Health in childhood and adolescence is a matter of contention. This is because it has consequences for the well-being of children and adolescents. Moreover, health problems have been linked to poor educational outcomes and have resulted in disadvantages throughout life [1].

Considerable international literature documents that poor infant health (commonly proxied by birthweight) is associated with lower educational achievement and attainment [2–5]. The studies mentioned herein made use of twin comparisons to demonstrate that the heavier twin of the pair would be more likely to attain a better adult outcome. Health problems in childhood and adolescence have also been shown to negatively influence educational outcome. Recent studies documented an association between childhood chronic health conditions and disadvantaged educational outcomes [6, 7]. Hyperactivity and conduct problems [8, 9], as well as depression [10–12], have also been associated with lower educational achievement. However, with respect to mental health problems, externalising problems such as hyperactivity and conduct problems have especially been found to impair educational outcomes [13, 14]. Moreover, Haas and Fosse [15] found that both mental and physical health in adolescence significantly affected educational performance and attainment. The research mentioned herein is in line with the social selection hypothesis which states that long-term negative consequences with respect to adult outcomes later result from poor early childhood health.

In contrast, the social causation hypothesis argues that socio-economic status (SES) affects health through a number of different mechanisms. According to Grossmann [16], SES can influence health through many pathways. Firstly, children’s health is influenced by material health inputs such as medical care, food and the quality of housing. Children with a higher SES might have access to better material health inputs due to better family finances. Secondly, parents with a higher SES might also adopt a healthier lifestyle (e.g., smoke and drink less) which, in turn, affects children’s health [13].

The direction of causality between health and SES is not entirely clear, neither theoretically nor empirically. Poorer health may be the result of a low SES. However, it could also be due to health selection, whereby poor health in childhood and adolescence influences educational attainment and adult SES, as discussed by Currie [17]. It is also possible that the relationship between SES and health is reciprocal. Social selection and social causation might operate simultaneously throughout the life course [18].

Previous research has shown that poor infant health (low birthweight) impairs health in childhood [19] and adolescence [20], and throughout the life course [1]. Therefore, we hypothesised that infant health has an impact on enrolment in higher education in early adulthood, both directly and indirectly, through adolescent health.

**Fig. 1:**
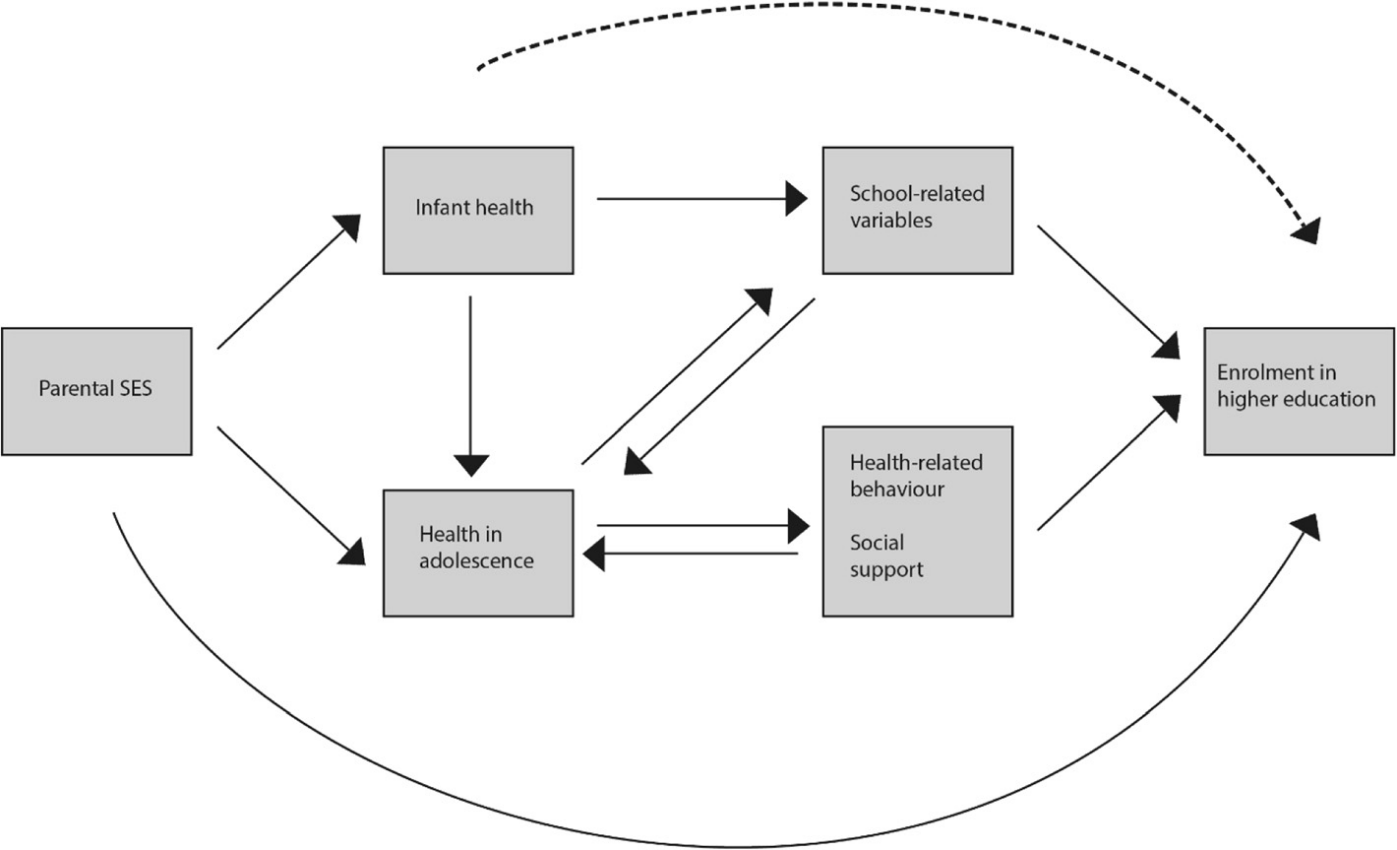
Conseptual model of the association between health and enrolment in higher education

Moreover, we assumed that parental SES is important in this regard. Children with a lower SES are likely to have a lower health status at birth [19, 21]. Highly educated mothers have been shown to adopt a healthier lifestyle during pregnancy [22]. In general, the circumstances of pregnancy and birth are better for mothers with a high SES than for those with a low SES. This might also be an interplay in this regard with genetic factors, which, in turn, affects the child’s health at birth [23]. Furthermore, living in a family with limited resources might affect child development and stress [24], which once again has consequences for child and adolescent well-being, health and educational outcome [25]. Therefore, we also hypothesised that the association between health at birth and that at adolescence and educational outcome is mediated by parental SES. Fig. 1 shows the analytic framework of the causal association between health and enrolment in higher education.

The purpose of this study was to examine the relationship between infant health, adolescent health and enrolment in higher education in early adulthood. We examined how infant and adolescent health act together with parental SES, health-related behaviour and academic factors to generate differences in the early life course with regard to later enrolment in higher education. The results of the present study highlight the trajectory of infant to adolescent health, and how health measured at different stages in life is associated with educational outcomes in early adulthood.

Norway provides an interesting context for analyses of the association between health and educational outcomes in early adulthood. Low infant mortality rates and the lowest proportion of low-birthweight babies, together with high enrolment by youths in higher education, have been reported in Nordic countries [26, 27]. Norwegian educational institutions are obliged to assist people with special needs so that most people are able to enrol at university or college. Consequently, the opportunity to obtain higher education for children with health problems in Norway is fairly high. However, when comparing different socio-economic groups, inequalities in young children with respect to mortality are not less pronounced in the Nordic countries than elsewhere in Europe [28]. This suggests that children with a low SES might be more disadvantaged, both in relation to health and educational opportunities.

## Methods

### Participants

Information from several linked data sources was used in this study, and included the Norwegian National Education Database; and The Historical Event Database, FD-Trygd; administered by Statistics Norway. These data sources offer rich longitudinal population data on income and wealth, welfare benefits and education, as well as demographic information whereby parents and children are linked. The Medical Birth Registry of Norway (MBRN) is also linked to a questionnaire survey on health, The Oslo Health Study 2000–2001 (UNGHUBRO). In 2000–2001, the survey was administered to all grade 10 pupils in Oslo, most of whom were aged 15–16 years. The overall response rate for the survey was 88 %. We included respondents born in Norway (*n* = 5 335). Compared with the national statistics obtained from the MBRN [29], the UNGHUBRO sample seemed to be fairly representative with respect to both birthweight and gestational length. All parents received written information about the questionnaire, and the students completed a consent form before participation. For youth less than 15 years of age the parents were contacted and asked to provide a separate informed consent form. The Regional Ethics committee, South East C approved the study.

### Measures

#### Outcome variable

Enrolment in higher education was the outcome variable, which was coded “1” if the individual entered higher education, and “0” otherwise. Any enrolment counted provided that the respondent was registered in higher education in October of any year between 2000 and 2011.

#### Infant health

Infant health was measured using birthweight and the breathing effort, heart rate, muscle tone, reflexes and skin color (Apgar) score. Birthweight and the five-minute Apgar score are obtained from the MBRN. Birthweight was entered into the models as a normal logarithm of birthweight. The five-minute Apgar score is an overall assessment of newborn well-being five minutes following delivery. A score of 7–10 is defined as normal [30]. Gestational length ranged from 23 to 47 weeks.

#### Self-rated health in adolescence

Self-rated health in adolescence was taken from the UNGHUBRO survey. The participants were asked: “What is your present state of health?, and they rated the current status of their own health on a four-point scale ranging from “very good” to “poor”. The self-rated health of the adolescents captured both the physical and psychological dimensions of well-being [31]. The survey also contained a battery of questions relating to psychological distress in adolescence which covered:

- Fear (suddenly feeling panicky for no reason).
- Suddenly feeling frightened or anxious.
- Feeling faint or dizzy.
- Feeling tense or harassed.
- Being self-critical (easily finding fault with oneself).
- Sleeplessness.
- Feeling depressed or dejected.
- Feeling useless and of little worth.
- Feeling that everything is a burden.
- Feeling hopeless about the future.

Psychological distress was measured by combining these 10 items into the Symptom Checklist (SC)-10 scale score – a validated 10-item short version of the original Hopkins Symptom Checklist (SCL-90) [32]. Low scores indicated low levels of psychological distress and high scores high levels thereof.

#### Parental socio-economic status

Parental SES was measured by separate variables for parental education, income and wealth, and was taken from the register data. Parental education constituted the education level of the parent with the highest education or of the only parent who was present. Parental education was divided into four levels: compulsory school or less, upper secondary school, Bachelor’s level and Master’s level or higher. The parental income and wealth variables were measured as both parents’ gross combined mean income during the years that the persons in the sample were aged 7–16 years. Income included salary, income from self-employment and state support benefits, e.g., unemployment, sickness and maternity. Wealth included taxable assets and financial capital. Parents’ income and parents’ wealth were originally recorded in the Norwegian currency (the Norwegian Krone). The logarithm of parental income and wealth was used in the analyses. These variables were also centred on their mean. It was determined whether or not participants lived with both parents by asking them the following question: “Who do you live with at present?” This variable was categorised into a “two-parent household”, and a “one-parent household” /“other household arrangement”.

#### Health-related behaviour

Physical activity was measured by asking: “During school hours, how many times a week do you take part in sport or participate in physical exercise to the extent that you feel out of breath or sweat?” Smoking habits were categorised into “non-smokers”, “previous smokers or occasional smokers”, and “regular smokers”. Alcohol consumption was measured by asking: “Have you every drunk so much alcohol that you became drunk?” Answers were coded into two categories of “no, never or once” and “2–3 times or more”.

#### Social support

Relationship to family was measured by asking: “When you think about your family, would you say that you feel attached to your family?” Relationships with friends were determined by the statement: “I feel closely attached to my friends”. Answers were coded according to two categories of “completely agree” and “otherwise”.

#### School-related variables

Grade points were calculated by combining the grades taken in grade 10 into 11 main school subjects (divided by 10 and centred around their mean). Future educational expectations were determined by the question: “What is the highest education that you have considered undertaking?” Answers were coded into two categories of “upper secondary school and lower” and “higher education”. Days absent from school due to illness were estimated by asking if the respondent had experienced pain or illness regularly over the last 12 months. The respondents were then asked if pain or illness had resulted in them having to stay home from school. The variable ranged from 0 to 10 days or more. Persons who reported no pain were coded as “0”.

#### Control variables

An immigrant background, gender and the study year were obtained from the register data and considered to be covariates, and used in all five models. Immigrant background was categorised as “native origin”, “non-Western second-generation immigrants” and “Western second-generation immigrants”.

### Analysis

The analyses of enrolment in higher education were performed using logistic regression, with odds ratio (OR) and 95 % confidence interval (CI). There were some missing values in the dataset (between six and 185 cases out of 5 354). Excluding missing cases or including the missing cases as a separate category could have led to a biased estimate. Therefore, multiple imputations were run using the mi impute chained command in Stata® 13. Five imputed datasets were used. The procedure replaced each missing value with a set of plausible values based on all other variables in the dataset. For further details, see White, Royston, Wood [33].

## Results

Table 1 lists the means and proportions for the whole sample across educational status. Of those who started in higher education, there was a higher proportion of women and a lower proportion of second-generation immigrants, compared to the group who did not commence with higher education. Moreover, Table 1 shows that birthweight, the Apgar score and general health in adolescence varied across the two educational groups, with a slightly higher birthweight and higher Apgar score and considerably better self-reported adolescent health in those who started in higher education. However, the psychological distress measure was quite similar across the two educational groups. Significantly higher parental SES was found in those who enrolled in higher education. A large proportion of this group lived with both parents and frequently reported feeling close to their family. Those who enrolled in higher education were less likely to smoke and participated in more physical activity than those who did not enrol in higher education. Alcohol habits and relationships with friends were very similar across the two groups. Respondents in the higher educational group reported higher academic aspirations, being absent from school less and obtaining higher marks in grade 10 (Table 1).

**Table 1:** Descriptive statistics^*^ for students who enrolled in higher education and for those who did not

*n* = observations	Did not enrol in higher education (*n* = 1 675)	Enrolled in higher education (*n* = 3 680)
Girls	39	54
Immigrant background		
Native background	71	77
Non-Western background	20	13
Western background	8	9
Health measures		
Log birthweight (mean)	8.12	8.14
Apgar score (mean)	9.04	9.11
Gestational length (mean)	39.6	39.6
Health in adolescence		
Poor	14	10
Good	56	55
Very good	28	35
Psychological distress (mean)	1.47	1.44
Parental education		
Primary school	23	6
High school	47	25
Undergraduate	22	38
Graduate	6	30
Parental wealth and income		
Wealth (log)	−0.56	28
Income (log)	−0.52	29
Two-parent household	57	72
Physical exercise (mean)	3.1	3.3
Smoking habits		
Never smoked	48	65
Quit or smokes sometimes	27	25
Smokes every day	24	10
Alcohol consumption		
Never or once	51	53
2–3 times or more	49	47
Social support		
Feel close to friends	67	69
Feel close to family	69	76
Expect to obtain a college education	27	67
Grades in grade 10	−0.65	27
Absence from school due to pain (mean)	1.8	1.5

*Apgar* breathing effort, heart rate, muscle tone, reflexes and skin color

*: Presented as a percentage unless otherwise stated

Table 2 shows the unadjusted single logistic regression analysis. It is demonstrated in Table 2 that all of the variables, excluding psychological distress, alcohol consumption and gestational length, were significantly associated with enrolment in higher education. Not surprisingly, good grades in grade 10, academic aspirations and parental SES seemed to be particularly important with respect to enrolment in higher education.

**Table 2:** Unadjusted single logistic regressions on enrolment in higher education

*n* = observations	Model, OR (95 % Cl)
	Single regressions, *n* = 5 354
Girls	1.84 (1.63–2.07)
Immigrant background	
Non-Western second-generation background (reference is a native background)	0.58 (0.50–0.68)
Western second-generation background	1.05 (0.84–1.30)
Health measures	
Birthweight (log)	1.45 (1.05–1.99)
Apgar score	1.15 (1.05–1.26)
Gestational length	1.00 (0.98–1.02)
Health in adolescence (reference is poor health)	
Good	1.45 (1.21–1.74)
Very good	1.80 (1.49–2.19)
Psychological distress (SCL-10)	0.90 (0.79–1.09)
Parental education (reference is primary school)	
High school	2.11 (1.75–2.56)
Undergraduate	6.7 (5.48–8.19)
Graduate	18.96 (14.62–24.52)
Parental wealth and income	
Wealth (log)	1.50 (1.30–1.70)
Income (log)	1.42 (1.28–1.58)
Two-parent household	1.97 (1.75–2.23)
Physical exercise	1.11 (1.06–1.16)
Smoking (reference is to never smoke)	
Quit or smokes sometimes	0.68 (0.59–0.78)
Smokes every day	0.30 (0.26–0.36)
Alcohol consumption (reference is never or once)	
2–3 times or more	0.92 (0.82–1.03)
Social support	
Feel close to friends	1.13 (1.00–1.28)
Feel close to family	1.43 (1.26–1.63)
Expect to obtain a college education	5.48 (4.81–6.23)
Grades in grade 10	6.18 (5.50–6.95)
Absence from school due to pain	0.86 (0.83–0.90)

*Apgar* breathing effort, heart rate, muscle tone, reflexes and skin color; *CI* confidence level; *OR* odds ratio; *SCL-10* Symptom Checklist-10 [taken from Hopkins Symptom Checklist (SCL-90)

To assess the relationship of infant and adolescent health and enrolment in higher education, multivariate logistic regression was applied in five different models (Table 3). Only significant variables from Table 2 were included in the multivariate analysis shown in Table 3.

**Table 3:** Adjusted logistic regression with regard to the impact of infant health and adolescent health on enrolment in higher education

*n* = observations	Model, OR (95 % Cl)				
	Model 1	Model 2	Model 3	Model 4	Model 5
	*n* = 5 354	*n* = 5354	*n* = 5 354	*n* = 5 354	*n* = 5 354
Gender					
Girls	1.85 (1.64–2.08)	2.00 (1.77–2.26)	2.22 (1.94–2.54)	2.52 (2.18–2.91)	2.03 (1.72–2.39)
Boys	Reference				
Immigrant background					
Non-Western	0.60 (0.51–0.70)	0.60 (0.51–0.70)	1.36 (1.09–1.68)	1.31 (1.05–1.63)	1.36 (1.08–1.73)
Western	1.05 (0.85–1.30)	1.057 (0.86–1.33)	0.88 (0.60–1.13)	0.89 (0.69–1.16)	0.83 (0.63–1.10)
Native	Reference				
Health measures					
Birthweight (log)	**1.43 (1.02**–**2.00)**	**1.46 (1.04**–**2.05)**	0.95 (0.65–1.40)	0.95 (0.64–1.40)	0.87 (0.56–1.35)
Apgar score	**1.12 (1.02**–**1.22)**	**1.11 (1.02**–**1.22)**	1.06 (0.95–1.17)	1.08 (0.97–1.15)	1.03 (0.91–1.18)
Health in adolescence					
Good		**1.56 (1.30**–**1.88)**	**1.45 (1.18**–**1.79)**	1.18 (0.95–1.46)	0.89 (0.70–1.14)
Very good		**2.17 (1.77**–**2.65)**	**1.87 (1.49**–**2.34)**	1.24 (0.97–1.58)	0.84 (0.63–1.10)
Poor	Reference				
Parental education					
High school			1.67 (1.34–2.09)	1.66 (1.25–1.34)	1.46 (1.13–1.87)
Undergraduate			4.68 (3.68–5.39)	4.66 (3.65–5.96)	3.11 (2.37-4.07)
Graduate			11.20 (8.28–15.16)	11.22 (8.25–15.25)	5.78 (4.16–8.07)
Primary school	Reference				
Parental wealth and income					
Wealth (log)			1.34 (1.24–1.44)	1.34 (1.25–1.44)	1.27 (1.17–1.37)
Income (log)			1.12 (1.06–1.17)	1.24 (1.06–1.18)	1.10 (1.04–1.16)
Two-parent household					
Yes			1.58 (1.37–1.82)	1.39 (1.20–1.61)	1.30 (1.10–1.53)
No	Reference				
Physical exercise				1.09 (1.04–1.16)	1.04 (0.98–1.10)
Smoking					
Quit or smokes sometimes				0.64 (0.54–0.75)	0.76 (0.63–0.91)
Smokes every day				0.34 (0.28–0.42)	0.57 (0.45–0.73)
Never smoke	Reference				
Feel close to friends					
Yes				0.89 (0.76–1.03)	0.80 (0.68–0.75)
No	Reference				
Feel close to family					
Yes				1.12 (1.08–1.48)	1.24 (1.04–1.48)
No	Reference				
Expect to obtain a college education					
Yes					2.64 (2.24–3.10)
No	Reference				
Grades in grade 10					3.45 (3.02–3.93)
Absence from school due to pain					0.89 (0.84–0.95)

*Apgar* breathing effort, heart rate, muscle tone, reflexes and skin color; *CI* confidence level; *OR* odds ratio

Bold values indicate when exposure shows significance

The odds of enrolment in higher education were higher for women (OR 1.85, 95 % CI: 1.64–2.08) than for men, and lower for youth from a non-Western immigrant background (OR 0.60, 95 % CI: 0.51–0.70) than for youth of native origin. The odds of enrolment in higher education increased with increasing birthweight (OR 1.43, 95 % CI: 1.02–2.00) and increasing Apgar score (OR 1.12, 95 % CI: 1.02–1.22) (model 1).

Very good general health in adolescence was associated with higher odds of enrolling in higher education (OR 2.17, 95 % CI: 1.77–2.65). After adjusting for health in adolescence (model 2), infant health remained significant and barely changed. There was no significant interaction effect between health (infant and adolescent) and parental SES (results not shown).

Students whose parents had attained a high educational level had higher odds of starting in higher education (OR 11.20, 95 % CI: 8.28–15.16) compared to those with parents who had obtained a primary education or less. High parental income (OR 1.12, 95 % CI: 1.06–1.17) and wealth (OR 1.34, 95 % CI: 1.24–1.34) were associated with increased odds of starting in higher education. Students who lived in a two-parent household had higher odds of starting in higher education (OR 1.58, 95 % CI: 1.37–1.82) than those whose parents lived apart. The impact of infant health on enrolment in higher education was reduced when adjusted for SES and became nonsignificant (model 3). Moreover, the association between adolescent health and enrolment in higher education became weaker after parental SES was included. However, adolescent health was still important with respect to enrolment in higher education when youth was compared with comparable parental SES.

Model 4 included health-related behaviour variables, such as smoking and physical activity, as well as feeling close to family and friends (social support). Physical activity also seemed to be positively associated with the odds of starting in higher education (OR 1.09, 95 % CI: 1.04–1.16). Daily smoking or smoking sometimes was associated with much lower odds of starting in higher education (OR 0.34, 95 % CI: 0.28–0.42) compared to the odds for those who didn’t smoke. The odds of starting in higher education were higher for youth who felt close to their family (OR 1.12, 95 % CI: 1.08–1.48). However, feeling close to friends did not have any significant association with the odds of starting in higher education. Self-perceived health was insignificant after the inclusion of health-related and social support variables.

Youth who planned on obtaining higher education at primary school had higher odds of starting in higher education in early adulthood than those who did not (OR 2.64, 95 % CI: 2.24–3.10). Obtaining good grades was associated with higher odds of starting in higher education (OR 3.45, 95 % CI: 3.02–3.93). Days absent from school decreased the odds of starting in higher education (OR 0.89, 95 % CI: 0.84–0.95) (model 5). In model 5 feeling close to friends was associated with lower odds of starting in higher education (OR 0.80, 95 % CI: 0.67–0.94).

## Discussion

Infant health measures, such as birthweight and Apgar score, had a positive association with enrolment in higher education in the unadjusted results. These findings are consistent with prior literature in which it has been demonstrated that persons with good infant health [2, 3] have better adult outcomes, including educational outcomes. However, after adjusting for parental SES, the relationship between infant health and enrolment in higher education became nonsignificant. The relationship seems to be caused by the fact that parental SES influences both infant health and educational enrolment. This finding is in line with previous research in which it was shown that parental SES influenced infant health [19, 21] and that parental SES predicted educational attainment [34]. The results in the present article do not support the notion that poor infant health, measured by birthweight and Apgar score, have a direct impact on enrolment in higher education, and thus contradicts previous research [2, 3]. The reasons for this could be owing to good opportunities for children with underprivileged health to participate in higher education in Norway, or the UNGHUBRO sample might have been positively selected. For example, the sample might not have included youth with the most serious health conditions who probably do not enrol in higher education. Moreover the differences could also reflect methodological differences. However, the results in the present study show that low-birthweight infants are less likely to enrol in higher education as adults, and that the negative association with low birthweight owing to low parental SES could have an adverse impact on enrolment in higher education.

Moreover, self-reported health by adolescents remains a significant and strong predictor of enrolment in higher education, even after adjusting for parental SES. Overall, students who had reported very good self-perceived general health during adolescence were more likely to enrol in higher education. These findings are in line with prior literature in which it has been demonstrated that there are better educational outcomes for people with good adolescent health [15]. However, the association between adolescent health and enrolment in higher education was reduced and became nonsignificant when the health behaviour of the adolescents and their relationship with their families were adjusted for.

The results imply that the association between adolescent health and educational outcome is mediated by health-related behaviour and the child’s relationship with his or her family in adolescence. It could be interpreted that health-related behaviour and support from family influences adolescent health, which, in turn, impacts on enrolment in higher education. Previous research has documented that health-related behaviour, such as physical activity [35] and smoking [36], affect adolescent health. Moreover, it has been documented in the literature that parental investment is crucial with respect to children’s educational attainment [37]. In the final model, feeling close to friends is associated with lower odds of enrol in higher education. This might reflects negative effects of over-investment in peers who do not support aspirations for higher education. Dyer [38] shows that relationships with friends that were focused on learning are positively related with academic outcomes, while relationships with deviant friends impair academic outcomes. Moreover, the present study indicated that living in a two-parent household was important with regard to the odds a child starting in higher education being increased. The present study did not find any evidence that parental SES mitigated the impact of poor health on educational outcome (the interaction effects between health and parental SES were insignificant), which is comparable with previous research [39]. Finally, the present study showed that future educational expectations and obtaining good grades in grade 10 were strong predictors of enrolment in higher education. Specifically, this association held for grades that were quite strong. Numerous studies have documented that good grades predict future educational success [14, 40]. The results in this study demonstrate that parental SES are the main driver for enrolment in higher education, followed by good grades in grade 10, future educational expectations and gender.

A strength of this study was the combination of survey data that provided information on health in adolescence, linked to register data on infant health and longitudinal details with respect to eduational outcomes in early adulthood. Information on both infant (Apgar score and birthweight) and adolescent health is often lacking in studies that focus on the relationship between health and educational attainment. In addition, this study contained rich information on parental SES (income, wealth and education). The survey was administered to grade 10 pupils in Oslo, and represented a large sample. The response rate was also high (88 %). The study sample was representative of youth aged 15–16 years.

A limitation of this study was that the measure of adolescent health was self-reported. The survey contained information on detailed health problems. However, in the present study, these variables did not have any significant association with enrolment in higher education. The study lacked data on diagnoses and objective information on health problems and illness in adolescence. The data used in this paper did not fully address the potential for omitted variables as an experimental identification strategy was not employed. However, this study adjusted for a wide range of family background and school-related variables which generally affect enrolment in higher education.

## Conclusion

There are lower odds of enrolment in higher education for infants of low birthweight. However, this result seems to reflect the fact that parental SES are associated with both infant health and enrolment in higher education. Adolescent health are associated with enrolment in higher education, even after adjusting for parental SES. However, a considerable proportion of this association seems to be attributable to health-related behaviour and the relationship of the adolescent with his or her family.

## Abbreviations

SES: Socio-economic status
MBRN: Medical Birth Registry of Norway
UNGHUBRO: The Oslo Health Study
OR: Odds ratio
CI: 95 % confidence interval
SC-10: Symptom Checklist
SCL-90: Symptom Checklist

## References

[1] Moster D, Lie RT, Markestad T (2008). Long-term medical and social consequences of preterm birth. N Engl J Med.

[2] Black SE, Devereux PJ, Salvanes KG (2007). From the Cradle to the Labor Market? The Effect of Birth Weight on Adult Outcomes. Q J Econ.

[3] Chatterji P, Kim D, Lahiri K (2014). Birth weight and academic achievement in childhood. Health Econ.

[4] Oreopoulos P, Stabile M, Walld R, Roos LL (2008). Short-, medium-, and long-term consequences of poor infant health: an analysis using siblings and twins. J Hum Resour.

[5] Saigal S, Hoult LA, Streiner DL, Stoskopf BL, Rosenbaum PL (2000). School difficulties at adolescence in a regional cohort of children who were extremely low birth weight. Pediatrics.

[6] Champaloux SW, Young DR (2015). Childhood chronic health conditions and educational attainment: a social ecological approach. J Adolesc Health.

[7] Maslow GR, Haydon AA, Ford CA, Halpern CT (2011). Young adult outcomes of children growing up with chronic illness: an analysis of the National Longitudinal Study of Adolescent Health. Arch Pediatr Adolesc Med.

[8] Currie J, Stabile M (2006). Child mental health and human capital accumulation: the case of ADHD. J Health Econ.

[9] Fletcher JM, Wolfe BL (2008). Child mental health and human capital accumulation: the case of ADHD revisited. J Health Econ.

[10] Fletcher JM (2010). Adolescent depression and educational attainment: results using sibling fixed effects. Health Econ.

[11] Jonsson U, Bohman H, Hjern A, von Knorring L, Olsson G, von Knorring AL (2010). Subsequent higher education after adolescent depression: a 15-year follow-up register study. Eur Psychiatry.

[12] Needham BL (2009). Adolescent depressive symptomatology and young adult educational attainment: an examination of gender differences. J Adolesc Health.

[13] Goodman J, Currie J (2010). Parental socioeconomic status, child health, and human capital. Int Encycl Educ.

[14] Sagatun A, Heyerdahl S, Wentzel-Larsen T, Lien L (2014). Mental health problems in the 10th grade and non-completion of upper secondary school: the mediating role of grades in a population-based longitudinal study. BMC Public Health.

[15] Haas SA, Fosse NE (2008). Health and the educational attainment of adolescents: evidence from the NLSY97. J Health Soc Behav.

[16] Grossman M (1999). The human capital model of the demand for health.

[17] Currie J (2009). Healthy, wealthy, and wise: socioeconomic status, poor health in childhood, and human capital development. J Econ Lit.

[18] Elovainio M, Ferrie JE, Singh-Manoux A, Shipley M, Batty GD, Head J (2011). Socioeconomic differences in cardiometabolic factors: social causation or health-related selection? Evidence from the Whitehall II Cohort Study, 1991–2004. Am J Epidemiol.

[19] McGovern ME. Still unequal at birth: birth weight, socio-economic status and outcomes at age 9. The Economic and Social Review 2013;44(1, Spring):32.

[20] Singh GK, Kenney MK, Ghandour RM, Kogan MD, Lu MC (2013). Mental health outcomes in us children and adolescents born prematurely or with low birthweight. Depression Res Treat.

[21] Currie J, Moretti E (2007). Biology as destiny? Short and long-run determinants of intergenerational transmission of birth weight. J Labor Econ.

[22] Kramer MS, Seguin L, Lydon J, Goulet L (2000). Socio-economic disparities in pregnancy outcome: why do the poor fare so poorly?. Paediatr Perinat Epidemiol.

[23] Rutter M (2007). Genes and behavior: nature-nurture interplay explained.

[24] Schilling EA, Aseltine RH, Gore S (2008). The impact of cumulative childhood adversity on young adult mental health: measures, models, and interpretations. Soc Sci Med.

[25] Fergusson D, Woodward L (2002). Mental health, educational, and social role outcomes of adolescents with depression. Arch Gen Psychiatry.

[26] OECD. Health at a Glance: OECD indicators. OECD Publishing. 2009.

[27] OECD. Education at a Glance. OECD Publishing. 2013.

[28] Popham F, Dibben C, Bambra C (2013). Are health inequalities really not the smallest in the Nordic welfare states? A comparison of mortality inequality in 37 countries. J Epidemiol Community Health.

[29] Health NIoP (2014). Medical Birth Registry of Norway, statistic bank.

[30] Green TP. Diagnostic approach to respiratory disease. In: Kliegman R, Nelson EW, editors. Textbook of pediatrics. 19th ed. Philadelphia: Saunders Elsevier; 2011.

[31] Boardman JD (2006). Self-rated health among U.S. adolescents. J Adolesc Health.

[32] Dalgard OS, Thapa SB, Hauff E, McCubbin M, Syed HR (2006). Immigration, lack of control and psychological distress: findings from the Oslo Health Study. Scand J Psychol.

[33] White IR, Royston P, Wood AM (2011). Multiple imputation using chained equations: issues and guidance for practice. Stat Med.

[34] Erikson R, Jonsson JO (1996). Can education be equalized? The Swedish case in comparative perspective.

[35] Strong WB, Malina RM, Blimkie CJ, Daniels SR, Dishman RK, Gutin B (2005). Evidence based physical activity for school-age youth. J Pediatr.

[36] Arday DR, Giovino GA, Schulman J, Nelson DE, Mowery P, Samet JM (1995). Cigarette smoking and self-reported health problems among U.S. high school seniors, 1982–1989. Am J Health Promot.

[37] Blau, Peter M. and Otis Dudley Duncan. 1967 The American Occupational Structure. New York: John Wiley and Sons. Duncan, Beverly.

[38] Dyer NE. The impact of close friends’ academic orientation and deviancy on academic achievement, engagement, and competence across the middle school transition. PhD thesis,Texas A&M University; 2010.

[39] Currie J, Hyson R (1999). Is the impact of health shocks cushioned by socioeconomic status? The case of low birthweight. Am Econ Rev.

[40] Winding TN, Nohr EA, Labriola M, Biering K, Andersen JH (2013). Personal predictors of educational attainment after compulsory school: influence of measures of vulnerability, health, and school performance. Scand J Public Health.

